# Nutraceuticals for Skin Care: A Comprehensive Review of Human Clinical Studies

**DOI:** 10.3390/nu10040403

**Published:** 2018-03-24

**Authors:** Almudena Pérez-Sánchez, Enrique Barrajón-Catalán, María Herranz-López, Vicente Micol

**Affiliations:** 1 Instituto de Biología Molecular y Celular (IBMC), Universidad Miguel Hernández (UMH), Edificio Torregaitán, 03202 Elche, Spain; almudena.perez@umh.es (A.P.-S.); mherranz@umh.es (M.H.-L.); vmicol@umh.es (V.M.); 2 Ilice Effitech, UMH Scientific Park, 03202 Elche, Spain; 3 CIBER: CB12/03/30038, Fisiopatología de la Obesidad y la Nutrición, CIBERobn, Instituto de Salud Carlos III (ISCIII), 07122 Palma Sola, Spain

**Keywords:** nutraceutical, skin, natural compound, polyphenols

## Abstract

The skin is the body’s largest organ, it participates in sensitivity and offers protection against microorganisms, chemicals and ultraviolet (UV) radiation. Consequently, the skin may suffer alterations such as photo-ageing, immune dysfunction and inflammation which may significantly affect human health. Nutraceuticals represent a promising strategy for preventing, delaying, or minimising premature ageing of the skin and also to alleviate certain skin disorders. Among them, bioactive peptides and oligosaccharides, plant polyphenols, carotenoids, vitamins and polyunsaturated fatty acids are the most widely used ingredients. Supplementation with these products has shown evidence of having an effect on the signs of ageing and protection against UV radiation ageing in several human trials. In this review, the most relevant human studies on skin nutraceuticals are evaluated and the statistical resolution, biological relevance of their results, and, the trial protocols are discussed. In conclusion, quality and rigorousness of the trials must be improved to build credible scientific evidence for skin nutraceuticals and to establish a cause-effect relationship between the ingredients the beneficial effects for the skin.

## 1. Introduction

**Figure 1:**
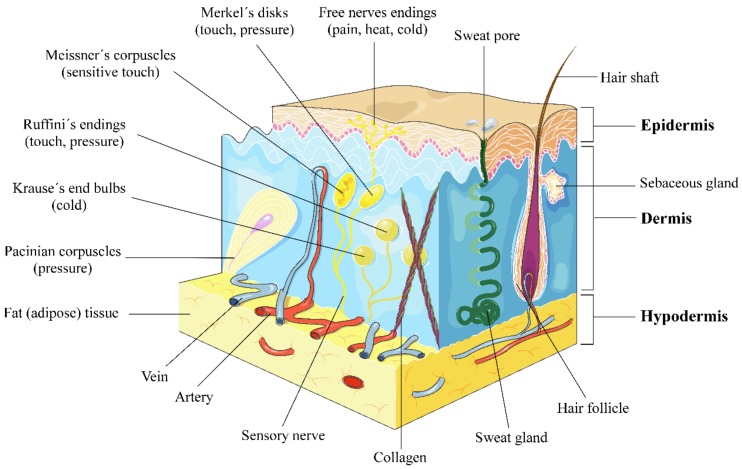
Human skin anatomy. There are three mechanoreceptor categories: tactile, proprioceptors and baroreceptors. The four major types of tactile mechanoreceptors are Merkel’s disks, Meissner’s corpuscles, Ruffini’s endings, and Pacinian corpuscles. The fifth type of mechanoreceptor, Krause’s end bulbs, is found only in specialised regions to detect cold. Free nerve endings are sensitive to painful stimuli, to hot and cold, and to light touch. This figure was created using Servier Medical Art [19], licensed under the Creative Commons Attribution 3.0 Unported License (www.creativecommons.org/licenses/by/3.0/).

The skin is the body’s largest organ, representing one sixth of the total body weight, and its main role is to act as a chemical and physical barrier to protect the body against harmful external environmental agents such as pathogen, ultraviolet (UV) radiation exposure, chemical threats, temperature changes and even dehydration [1,2,3]. The skin is composed of three main layers with different underlying structures: (a) the epidermis, (b) the dermis and (c) hypodermis or subcutaneous tissue [4] (Figure 1).

The epidermis, of ectodermal origin, is the major protective outer layer and serves as the body’s point of contact with the environment. The stratum corneum is the outermost layer of the epidermis, consisting of dead cells or corneocytes and has a thickness between 10 μm and 30 μm. Underneath the stratum corneum, are living keratinocytes, melanocytes and Langerhans cells [2,3,5]. Keratinocytes are the predominant cell type in the epidermis producing keratin, a protein that makes the skin waterproof. Another significant cell group in the epidermis is that composed of the melanocytes. These cells form a heterogeneous group of cells in the human body and are present in the epidermis–dermal junction and hair follicles. Melanocytes produce melanin, a pigment responsible for skin pigmentation and photoprotection. Melanin may have other important physiological effects, including regulatory influence of epidermal homeostasis, free radical scavenging to protect against oxidative stress and even antimicrobial activity [6,7,8,9,10,11,12]. Langerhans cells are immune dendritic cells protecting against external substances and microorganisms [13,14,15].

The dermis, of mesoderm origin, is the layer that provides strength and elasticity to the skin. It includes the vascular, lymphatic and neuronal systems. It also contains sweat pores and hair follicles (Figure 1). The dermis is primarily composed of complex extracellular matrix (ECM) proteins, specially collagen fibres. ECM proteins can be categorised as either structural such as collagen and elastin or non-structural (glycoproteins), depending on their function. Integrins are essential compounds of the ECM, in addition to a group of matrix metalloproteinases (MMPs) and growth factors (GFs) [16,17]. Dermal collagen represents the most abundant ECM protein constituting 90% dry weight of the skin. Dermal connective tissue collagen is responsible for the skin’s tensile strength and mechanical properties [18]. The dermis also contains abundant immune cells and fibroblasts (the major cell type present in this layer), which are involved in the synthesis of many of the ECM components. Blood vessels which provide nutrients to the skin and help regulate body temperature are also present in the dermis.

The hypodermis or subcutaneous tissue helps insulate the body from heat and cold, provides protective padding and serves as an energy storage area.

**Figure 2:**
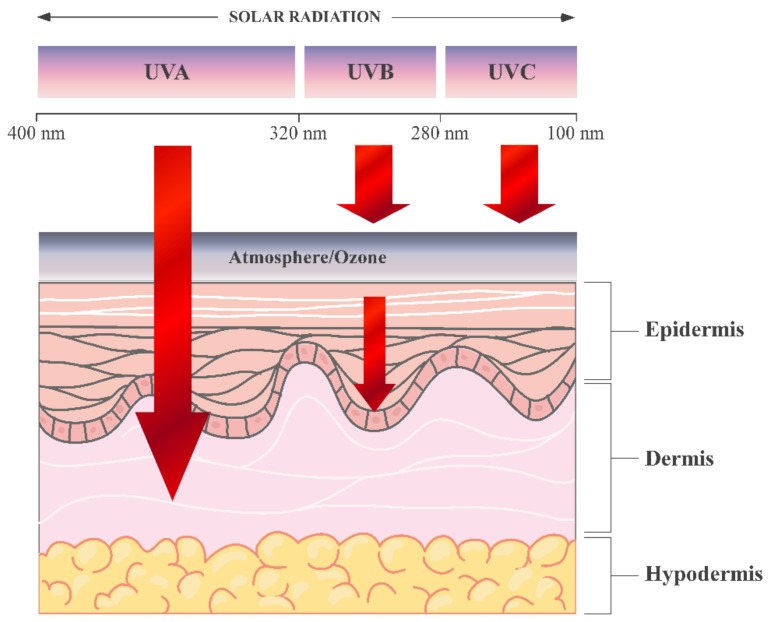
UV penetration into the layers of the skin. The figure was created using Servier Medical Art [19], licensed under the Creative Commons Attribution 3.0 Unported License (www.creativecommons.org/licenses/by/3.0/).

UV exposure is a major causative factor for age-related changes including inflammation, degenerative ageing, ECM degeneration and cancer. UV radiation is divided into long wave UVA (320–400 nm), medium wave UVB (280–320 nm), and shortwave UVC (100–280 nm), which is absorbed by the ozone layer. UVB represents only 0.3% of the sun’s emission reaching the ground, and UVA radiation reaches Earth surface almost entirely. UV penetrates the skin in a wavelength-dependent manner. UVA (longer wavelength) reaching the dermis and UVB is almost entirely absorbed by the epidermis [20] (Figure 2). 

**Figure 3:**
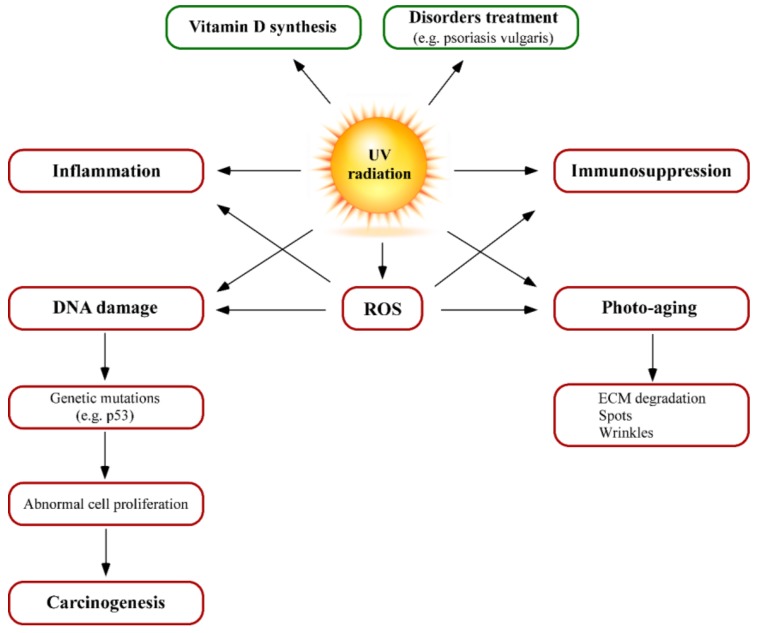
Summary of UV irradiation effects on the skin: positive (green) and adverse effects (red).

Solar UV radiation can interact with many molecules (chromophores) in different layers of the skin. These interactions may have both positive and negative biological effects, depending on wavelength, radiation exposure and UV sources. The positive effects mainly include vitamin D synthesis and treatment of different skin disorders (Figure 3). It is well known that solar radiation promotes the synthesis of vitamin D precursors at the skin. Most people can synthesise enough vitamin D from being out in the sun daily for short periods with their forearms, hands or lower legs uncovered. However, some populations as Africans, African/black Americans, or those from low sun-exposed areas, cannot synthesise it from limited sun exposure. In these situations, dermatologists recommend to get vitamin D from diet or vitamin supplements rather than from extra-exposure to UV.

The UVB photons enter the skin and photolyse 7-dehydrocholesterol to previtamin D_3_, which in turn, is isomerized by body’s thermal energy to vitamin D_3_. Deficiency of vitamin D causes growth retardation in children and can precipitate and aggravate osteoporosis and osteopenia in adults. However, this is only associated with extremely low sun exposure lifestyles (northern Native people or some Asian populations). In addition, phototherapy is also an option in the treatment of many skin pathologies such as psoriatic and nonpsoriatic (e.g., morphea, scleroderma, vitiligo, atopic dermatitis) disorders [21,22,23].

The adverse effects of UV-radiation include inflammation, immunosuppression and/or allergy disorders, UV-increased sensitivity by drugs (corticoids), photo-aging, DNA damage, oxidative stress and carcinogenesis (Figure 3). UV-mediated skin inflammation is externally characterised by sunburn or erythema. This situation can be visually identified by skin redness or erythema, which is due to blood flow increase caused by dilation of the superficial blood vessels in the dermis. High UV doses can result in oedema, blistering, peeling and pain after exposure and UVB radiation, which is more erythmogenic than UVA radiation [24]. UVB induces a cytokine cascade, neuroactive and vasoactive mediators in the skin, resulting in inflammatory responses. If the dose of UV exceeds a certain threshold, affected keratinocytes respond by activating an apoptotic pathway. Such cells can be identified by their pyknotic nuclei and are known as “sunburn cells” [25,26,27,28,29]. From a molecular point of view, damage signals such as p53 activation significantly alter keratinocyte physiology mediating cell cycle arrest and activating DNA repair. NF-κB nuclear transcription factor is also activated by UV radiation, leading to the initial steps in the inflammatory process of sunburn reactions that increases the expression of proinflammatory cytokines interleukin (IL)-1, IL-6, tumor necrosis factor (TNF)-α and vascular endothelial growth factor (VEGF) [30].

UV-induced immunosuppression does not affect only an irradiated area but also influences the whole immune system as skin includes all the cell types also present in secondary lymphoid organ such as the spleen, lymph nodes and tonsils [31]. The main cells affected by UV radiation are Langerhans cells and T lymphocytes. Langerhans cells are dendritic cells critical for the presentation of antigens to the immune system. Langerhans cells are located in the epidermis and are responsible for T-lymphocyte activation in response to foreign antigens. Several studies have demonstrated that UVB radiation alters the number of Langerhans cells (decrease density), morphology and immunological function. Also, UVB radiation has been shown to induce T cell tolerance via modulation of the function of antigen-presenting cells like dendritic cells, leading to immunosuppression [32].

Skin ageing is a complex biological process resulting from two synergistic mechanisms: intrinsic and extrinsic factors. On the one hand, intrinsic or endogenous ageing, is an unavoidable phenomenon that includes several factors such as cellular metabolism, genetics, hormone and the passage of time. It is clinically associated with increased fragility and loss of elasticity. On the other hand, extrinsic or exogenous ageing can be avoided and is caused by repetitive exposure of the skin or body to harmful agents, especially UV light (photo-aging), inappropriate diet, pollution, chemicals and toxins [33,34]. UV radiation increases matrix metalloproteinases (MMPs) expression in human skin. MMPs are responsible for degrading ECM proteins such as collagen, fibronectin, proteoglycans and elastin (functional support). In addition, MMPs play an important role in carcinogenesis affecting several processes related to tumour progression such as growth, angiogenesis and metastasis [35,36,37]. Therefore, photo-aging is characterised by a disturbed equilibrium in the accumulation and degradation of ECM, losing elasticity, irregular pigmentation, dryness and wrinkling [38,39,40,41,42,43,44,45,46]. Wrinkling and pigmentation are also directly associated with premature photo-aging and are considered the most critical skin events [47].

UV radiation overexposure causes generation of reactive oxygen species (ROS), leading to an oxidative stress status [48,49]. This prooxidative situation has relevant consequences in cell homeostasis such as lipid and protein oxidation, loss of mitochondrial potential and DNA damage. In addition, ROS increase other UV effects such as DNA damage, inflammation and ageing, as they can activate inflammatory responses and up-regulate matrix metalloproteinase (MMP) production and activity, resulting in collagen breakdown. Skin spontaneously responds to high ROS levels by activating detoxifying enzymes such as superoxide dismutase (SOD), catalase (CAT), thioredoxin reductase (TrxRs) and using other antioxidant molecules such as glutathione (GSH), α-tocopherol (vitamin E) and ascorbic acid (vitamin C). However, this response may not be effective enough to prevent oxidative damage of cutaneous cells after exposure to carcinogenic agents [50,51] (Figure 3).

UV radiation is only a fraction of the solar radiation; however, it is responsible for most of its carcinogenic activity as UV photons can affect the DNA integrity, homeostasis and induce mutations of genes including oncogenes and tumour suppressor genes. Although UVB represents a minority part of the whole radiation that reaches to the ground, it is the most dangerous and genotoxic component of sunlight, affecting nucleic acids in the epidermis [52,53]. UVB results in the formation of cyclobutane pyrimidine dimers (CPDs) and pyramidine-pyrimidone photodimers that may lead to DNA mutations and cancer [38,39,54]. However, UVA direct effect over nucleic acids is scarce, being its genotoxic effect mainly mediated by ROS as described above.

**Figure 4:**
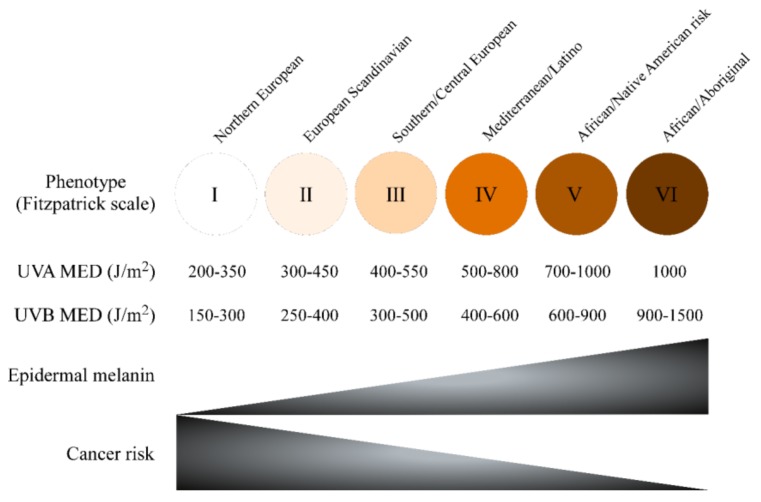
Influence of pigmentation and phototype on skin cancer risk.

Skin complexion is among the most critical factors of UV sensitivity and skin cancer risk. The “Fitzpatrick Scale”, developed in the 1970s by Dr. T.B. Fitzpatrick, is a semi-quantitative scale made up of six pigmentation categories (phototype) that describe skin colour by skin pigmentation and sensitivity to UV radiation. Minimal Erythematous Dose (MED) is a quantitative method to report the amount of UV, particularly UVB, needed to induce sunburn in the skin 24–48 h after exposure by determining erythema (redness) and oedema (swelling) as endpoints. Low Fitzpatrick phototype correlates with both MED and with melanoma and other skin cancer risk [55,56] (Figure 4). 

Besides biological skin function, the skin plays a role in physical attractiveness. Skin appearance is determined by its texture, colour, and different characteristics such us elasticity, sweat and sebum production. It is accepted that nutritional status concerning both macro and micronutrients is important for skin health and appearance [57]. For example, dermatological signs of vitamin C deficiency include skin fragility, alterations in hair texture as corkscrew hairs and bleeding gums as well as impaired wound healing [58,59,60,61,62,63,64].

Food and cosmetic industries are developing new strategies to establish the relation between nutrients consumption and skin health. Consequently, the use of food ingredients and supplements that claim to reduce the risk of skin disorders or alleviate skin ageing is increasing [65]. Dietary supplementation with vitamins, minerals or essential fatty acids is proposed to improve skin conditions [65]. Most of the bioactive food compounds responsible for the positive effects on health are predominantly derived from plants while a few are derived from animal sources [66]. 

The term “nutraceutical” is derived from the combination of “nutrition” and “pharmaceutical” and was established by Stephen DeFelice in 1989, founder and chairman of the Foundation for Innovation in Medicine (FIM), Cranford, New Jersey [67,68]. According to DeFelice, nutraceutical can be defined as “a food (or a part of it) that provides medical or health benefits, including the prevention and/or treatment of a disease”. Nutraceuticals may be used to improve health, delay the ageing process, prevent chronic diseases such as obesity, increase life expectancy, or support the structure or function of the body. 

Nowadays, nutraceuticals have received considerable interest due to their nutritional potential, safety and therapeutic effects. Nevertheless, although nutraceuticals have shown promising results in various complications, their uncontrolled use may not be devoid of secondary effects so they should be strictly regulated as prescription drugs.

The nutraceutical industry’s main segments include dietary supplements, herbal/natural products and functional foods [69]. Nutritional supplements and herbal/nature products are the most rapidly growing segments. A recent market research (Variant Market Research, Pune, India), proposed that the global nutraceuticals market is expanding and would reach $340 billion by 2024, growing at a Compound Annual Growth Rate (CAGR) of 7.2% from 2016 to 2024. Several market factors have been related to the significant growth of this sector: increasing healthcare, growing popularity for nutrition and rising demand for nutraceuticals [70].

Nutraceuticals can be classified by several criteria: food source, mechanism of action, chemical nature and specific benefit for health. They may be macronutrients (nutrients salts/chemicals elements required in large ammounts, e.g., potassium, magnesium, calcium and omega 3 acids), micronutrients (nutrients salts/chemicals elements required in small quantities, e.g., vitamins and minerals) and phytochemicals. Food sources used as nutraceuticals are all natural and can be categorised as: dietary fiber, prebiotics, probiotics, polyunsaturated fatty acids, minerals, amino acids and peptides, carotenoids, vitamins, phytochemicals and spices [69,71]. 

In this review, published data on the effects of nutrients on human skin are summarised. The design of the studies and the results of the different human trials that use prototype or commercial nutraceutical products are discussed to establish a cause-effect relationship between the consumption of the ingredient and the skin effects. The studies are organized according to the different families of ingredients used in the dietary supplements.

## 2. Bioactive Peptides

Peptides are short polypeptidic chains formed by few amino acids and with a low molecular weight, usually under 3 kDa. Since some of them carry out critical biological activities, these are generally known as bioactive peptides. Peptides used for cosmetic purposes are typically derived from collagen with improved bioavailability and solubility compared to the whole protein. Bioactive peptides have been used in several nutraceutical formulations claiming antiageing and skin reaffirming properties as described below.

In a double-blind placebo-controlled study, 114 subjects received 2.5 g of bioactive collagen peptide (BCP) VERISOL^®^ or placebo per day for 8 weeks, 57 subjects in each treatment group. Skin wrinkles were measured before the treatment and after 4 and 8 weeks. The intake of BCP promoted a statistically significant reduction of eye wrinkle volume compared to placebo group after 4 and 8 weeks of treatment. In addition, BCP intake showed a statistically significant higher content of procollagen type I and elastin. Moreover, an increased fibrilin content was detected after BCP treatment, however this increase was not statistically significant. Authors concluded that oral intake of VERISOL^®^ significantly reduced skin wrinkles and had positive effects on skin matrix synthesis (*p* < 0.05) [72]. 

In a placebo-controlled clinical trial, 60 women were screened, 33 subjects received a placebo or one of two treatments: a specific mixture of collagen peptides of fish origin (Peptan^®^F) or porcine origin (Peptan^®^P). Subjects took a formulated drink that contain either 10 g Peptan^®^ or 10 g placebo for 56 days. Facial skin parameters were measured after 4, 8 and 12 weeks of treatment. Oral intake of Peptan^®^F after 8 weeks of treatment showed a significant increase of skin moisture (12%), while Peptan^®^P increased skin moisture by 28% after 4 weeks of treatment and minimised skin micro-relief. In another double-blind placebo-controlled study, 106 women were randomly allocated to either a placebo or Peptan^®^F group. This supplement was shown to reduce collagen fragmentation by 31% after 12 weeks of oral treatment, contributing to an anti-ageing effect [73]. The two fore-mentioned studies included a high number of subjects that provided enough statistical potency to observe statistically significant differences (*p* < 0.05).

**Figure 5:**
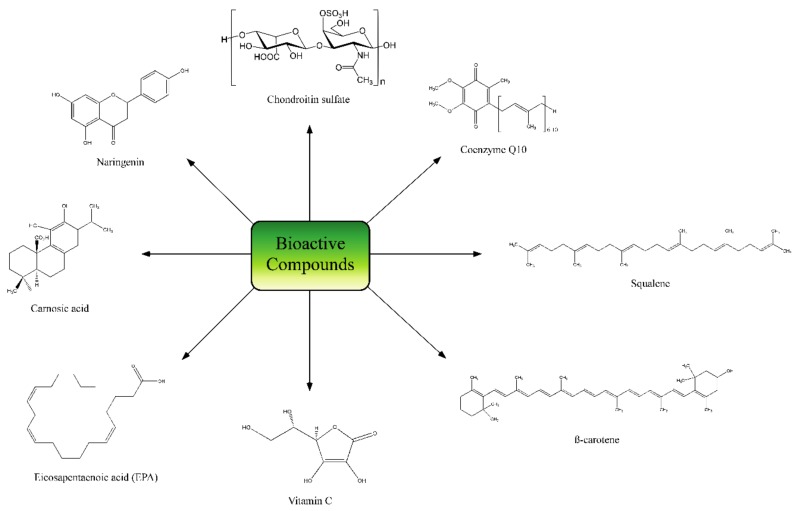
Representative structure compounds of different supplementation products.

In a recent clinical-laboratory study, 41 subjects received CELERGEN^®^, a nutraceutical product containing 570 mg marine collagen peptides (MCPs) derived from deep sea fish, 10 mg grape skin extract, 10 mg Coenzyme Q10 (CoQ10) (Figure 5), 10 mg luteolin and 0.05 mg Se of plant origin for 2 months of pre-treatment followed by 2 months of treatment. Supplementation improved skin elasticity, dermal ultrasonic markers and sebum production but no change was observed in several oxidative stress markers. Although the number of subjects was small, differences were statistically significant (*p* < 0.05). Authors concluded that a combination of MCPs with plant-derived antioxidants could be an effective and safe supplement to improve skin properties without risk of oxidative damage [74]. 

## 3. Bioactive Polysaccharides

Polysaccharides are sugar polymers with both structural and energy storage functions. They are present in plants, animals, fungi and procariota organisms with different structures, monosaccharide compositions and physicochemical properties. As occurred with bioactive peptides, a similar qualifier is used for those polysaccharides that exert biological activities, but in this case, with the peculiarity that it is reserved for those acting in a different organism than in which they were synthetised. Glycosaminocans, especially from marine origin, are the most used for nutraceutical formulations. They are based on an unbranched repeating disaccharide unit of an amino-sugar (*N*-acetylglucosamine or *N*-acetylgalactosamine) and an uronic acid (glucuronic or iduronic acid). The most relevant human trials are explained below.

Imedeen^®^ Derm One^®^ is a dietary supplement for skin care containing protein fractions and some glycosaminoglycans extracted from marine fish. In addition, this supplement contained vitamin C and zinc gluconate, both relevant ingredients for skin health. This product was used in a trial where 10 women were treated with 500 mg per day of Imedeen^®^ for 90 days. Evaluated parameters included wrinkles, mottles, dryness and brittleness of hair and nails. After 90 days of treatment, all signs were improved, and clinical observations were confirmed by changes in skin elasticity and thickness [75]. 

On the other hand, Vivida^®^ is a commercially available product containing different active polysaccharides derived from marine fish cartilage. The efficacy and safety of Vivida^®^ and Imedeen^®^ were compared in a double-blind study, in which 15 women were treated with 500 mg of Vivida^®^ per day, and 15 women received 380 mg of Imedeen^®^ per day for 90 days. Both treatments showed statistically significant improvements in skin conditions (epidermal and dermal thickness, elasticity and erythemal index) but Vivida^®^ was more effective than Imedeen^®^ for all parameters [76]. Nevertheless, the low number of subjects in the two previous studies compromise the consistency of the results. In another study with 144 healthy Caucasian subjects that provided significant statistical power, the subjects received Imedeen^®^ tablets containing 105 mg proteoglycan, 30 mg vitamin C and 15 mg zinc gluconate for 12 months. After treatment, Imedeen^®^ showed significant improvement in reduction of fine lines and overall photo-aging, in self-evaluation of skin condition, density, trans-epidermal water loss and skin smoothness. However, the long duration of the intervention study, i.e., 12 months, can raise several concerns. For marketing purposes, the consumer needs to denote positive effects within a reasonable period that may be around 30–60 days, otherwise, the consumer will stop taking the supplement. On the other hand, such a long period of consumption may elicit some safety concerns [77].

Another randomised, double-blind, placebo-controlled clinical study was performed with the ingredient Imedeen^®^. The protocol was well-designed and also provided enough statistical power (*p* < 0.05). 90 healthy women received 2 tablets of Imedeen^®^ or placebo daily. Total daily dose contained 210 mg Imedeen^®^ marine complex, 57 mg tomato and grape seed extracts and 60 mg vitamin C for 4 months. The clinical grading of overall facial appearance improved for both treatment groups, however, the degree of improvement was significantly higher for subjects treated with the anti-ageing skin supplement. Results showed a positive effect on the appearance of skin, decreasing periocular wrinkling, visual and tactile roughness and mottled pigmentation [78]. 

In a quasi-experimental clinical study lacking a placebo group, 47 male subjects received two tablets of Imedeen^®^ Man.Age.Ment containing per tablet: 105 mg marine protein, 27 mg vitamin C, 13.7 grape seed extract, 2 mg zinc, and 14.38 tomato extract for 6 months. Results showed significant improvements from baseline in skin hydration, dermal ultrasound density and reduction of skin pH (*p* < 0.05). The photographic assessment showed an improvement in the overall appearance and objective measurements correlated subject’s satisfaction by an increase of collagen and elastic fibers [79].

In a double-blind, randomised, placebo-controlled study, 84 Asiatic healthy subjects were divided into two groups receiving control and galacto-oligosaccharides (GOS) respectively. Subjects received GOS (1 g in a capsule) twice a day for 12 weeks. Results showed that the increase in corneometer values from baseline to week 12 was significantly higher in the GOS treated group and the transepidermal water loss (TEWL) was reduced significantly (*p* < 0.05). In addition, the differences in total and percentage of wrinkle areas between the two groups were significant after 12 weeks of GOS treatment [80]. The evidence derived from the study supports a substantial improvement of the skin condition (hydration and skin barrier function) in individuals 50 years of age. However, the rationale and the mechanistic aspects of how oligosaccharides exert that action need to be further explored, at least in cell models.

## 4. Bioactive Botanical Extracts

**Figure 6:**
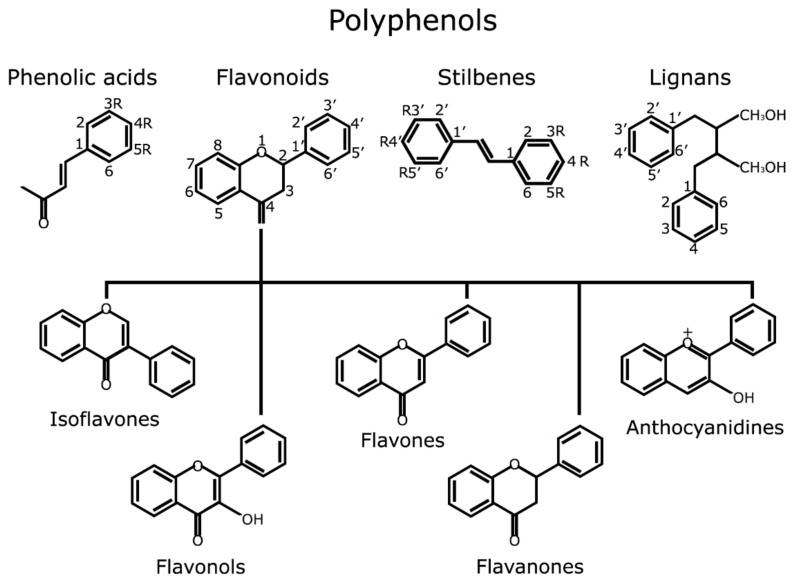
Main classes of polyphenols by structural classification. This image has been created and previously used by author in [91] and is under Creative Commons by Attribution (CC-BY) license (http://creativecommons.org/licenses/by/4.0/).

Botanical extracts are complex mixtures of natural compounds with different structures and origins. Their use in cosmetics and skin care is well known since ancient times and have been extensively reviewed before [81,82]. Polyphenols are the main natural compounds with cosmetic applications and include a large variability of different structures and families (Figure 6). Composition and proportion of polyphenols may greatly vary depending on plant family and extraction procedure. The following are the most relevant studies of nutraceutical products based on botanical extracts that have been tested in humans.

Pycnogenol^®^ is a standardised extract of bark of the French maritime Pine bark (*Pinus pinaster*) rich in flavonoids, such as catechins and procyanidins (B1, B2, B3, B7 C1 and C2), and phenolic acids such as caffeic, ferulic, and *p*-hydroxybenzoic acids. Pycnogenol^®^ and it has been demonstrated to have various biological and health effects such as cardiovascular and cholesterol lowering benefits and antioxidant, antidiabetic and anti-inflammatory activity [83,84,85,86]. Studies have proposed that Pycnogenol^®^ is highly bioavailable and the mixture is more effective than individual components (synergistic effect). However, due to low procyanidins absorption in the small intestine and its conversion into smaller flavan-3-ols derivatives by microbiota, the bioavailability in humans is still an unresolved issue. Pycnogenol^®^ oral supplements have been used in 21 Caucasian volunteers (1.1 mg/kg body weight). Results showed the photoprotective effect of this formulation against UV-light induced skin damages [87]. In other study, 20 healthy women were supplemented with three 25 mg tables of Pycnogenol^®^ (75 mg total) for 12 weeks. Pycnogenol^®^ intake showed a significant increase in the mRNA expression of hyaluronic acid synthase (HAS-1) and in gene expression involved in collagen de novo synthesis. Externally, Pycnogenol^®^ supplementation significantly improved hydration and elasticity of skin (*p* < 0.05) [88]. Nevertheless, the low number of subjects in the two previous studies raise doubts about the consistency of the results.

In a double-blind and placebo-controlled study, 62 women were treated with two Evelle^®^ tablets twice a day for 6 weeks. One Evelle^®^ tablet contains 10 mg Pycnogenol^®^, 30 mg vitamin C, 50 mg vitamin E, 75 μg biotin, 25 μg selenium, 7.5 mg zinc as gluconate, 50 mg bio-marine complex, 40 mg horsetail herb extract, 15 mg blueberry extract and 34 mg tomato extract. At the end of study, skin elasticity was found to be statistically significantly increased compared with placebo group. Skin roughness also was shown to be statistically significantly lower compared with the control group after 12 weeks of supplementation [89]. Further, 30 women with melasma were treated with one 25 mg tablet of Pycnogenol^®^ three times a day for 1 month (75 mg per day). The subjects were evaluated and clinically parameters such as melasma area index and skin pigmentation at the end of treatment, the melasma area and the average pigmentary intensity of the subjects showed a statistically significant decrease. The study conclude that Pycnogenol^®^ is therapeutically effective and safe in patients with melasma [90]. The differences observed were statistically significant (*p* < 0.001) but the number of patients was moderate and no placebo group was included.

Oral administration of a different French maritime pine bark extract called Flavangenol^®^, improved the clinical symptoms in photoaged facial skin. This oral supplement was administered to 112 healthy women under 60 years with age spots and multiple symptoms of photo-damaged skin (mottled pigmentation, roughness, wrinkles and swelling). 24 women were treated with 100 mg per day for 12 weeks while 88 women were treated with 40 mg per day for 24 weeks. At the end of the low-dose study, 24 subjects (out of 88) were treated with 40 mg per day for an additional 24 weeks to evaluate the long-term efficacy and safety of pine bark extract. In both time courses of 100 mg and 40 mg per day, a significant decrease in clinical grading of skin photo-aging scores such as reduction in the pigmentation of age spots was observed [92]. 

A randomised, double-blind, placebo-controlled study was performed to evaluate the effect of *Aloe* sterol supplementation on skin parameters such as elasticity, hydration and the collagen score. 64 healthy women were randomly divided to receive either a placebo or an *Aloe* sterol supplemented yogurt for 12 weeks. Results showed significance differences in skin moisture, TEWL, elasticity and collagen score between treatment and placebo groups. At the end of supplementation, elasticity and collagen content increased significantly with *Aloe* sterol intake [93]. In a similar study, 48 healthy men received *Aloe* sterol for 12 weeks. Results showed that treatment increased melanin index and elasticity while skin moisture decreased [94]. The two previous studies showed statistically significant differences (*p* < 0.05) and had a moderate number of subjects.

NutroxSun^®^, is a standardised formulation of citrus extract, obtained from immature grapefruit (*Citus paradisi*, enriched in citrus bioflavonoids as naringenin, Figure 5) and rosemary extract (*Rosmarinus officinalis*, enriched in phenolic compounds and diterpenes such as carnosic acid, Figure 5). To test the photoprotective efficacy of the combined extract, a human intervention study was performed by oral administration to volunteers for whom the minimal erythema dose (MED) was determined after exposure to UV radiation. At the 85th day of treatment, a significant increase in MED was observed (56%), indicating that more extended oral treatments can improve UV protection effects [95]. This study provided statistically significant differences in MED between placebo and intervention groups (*p* < 0.05) but the number of subjects was low. In addition, NutroxSun^®^ orally supplement has been used in 90 Caucasian female subjects to investigate the anti-inflammatory, photoprotective and anti-ageing effects of this combination. After 2 weeks of product consumption, results showed decreasing wrinkle depth and increasing elasticity at 100 and 250 mg extracts dose regimens. At 2 weeks of product use, results showed decreasing wrinkle depth and increasing elasticity at 100 and 250 mg extracts dose regimen [96]. In this second study, number of subjects was high and statistically significant differences were confirmed in MED and also in wrinkle depth, elasticity and skin lipoperoxides between placebo and intervention groups.

The skin photo-protective and anti-ageing effects of a powder extract of red orange fruit (Red Orange Complex^®^) were also reported in humans. The dietary supplement contained 2.8–3.2% *w*/*w* anthocyanins, 1.8–2.2% *w*/*w* hydroxycinnamic acids, 8.5–9.5% *w*/*w* flavone glycosides and 5.5–6.5% *w*/*w* ascorbic acid (see Figure 5 for representative structures). A moderate number of Caucasian subjects (20) received 100 mg of extract per day for 15 days. In the evaluation of skin erythema induced by UV irradiation the results showed that red orange extract intake significantly decreased UV-induced skin erythema (*p* < 0.05). In the evaluation of skin appearance homogeneity, subjects were exposed to solar lamp, and age spot pigmentation decreased 20%. The authors postulate that red-orange extract supplementation can present antioxidant activity and improves skin appearance and pigmentation [97].

Another study compared the effect of *Polypodium leucotomos*/Pomegranate combination (PPmix^®^) versus *Polypodium leucotomos* alone (Fernblock^®^) on skin biophysical parameters. Both extracts contained different polyphenols specifically ellagitannins such as punicalagin. 40 subjects (20 males and 20 females) received 480 mg PPmix^®^ or Fernblock^®^ per day for 3 months. Six skin parameters were measured: skin sebum, hydration, transepidermal water loss (TEWL), melanin index, erythema index and elasticity. After treatment, elasticity and hydration were improved and TEWL was reduced in both groups. Both treatments reduced erythema index but PPmix^®^ was more effective. Melanin index and skin sebum were decreased only by PPmix^®^. Melanin index and skin sebum were reduced only by PPmix^®^ [98]. A similar *P. leucotomos* extract was also utilized alone in Heliocare^®^ formulation. A randomised, double blind, placebo-controlled study was developed. Subjects from both sexes and with Fitzpatrick skin types I to IV were randomised to receive 240 mg of *P. leucotomos* extract (*N* = 20) or placebo (*N* = 20) twice a day for two months. The results confirmed *P. leucotomos* extract safety and showed that extract increased MED and reduced ultraviolet-induced erythema intensity (*p* < 0.05) [99].

## 5. Carotenoid Supplementation

Carotenoids are naturally occurring pigments with a lineal tretraterpenoid structure. They can be found in algae, photosynthetic bacteria and plants [100] providing red, orange and yellow colouration. Humans incorporate them from fruits and vegetables sources, with α-carotene, β-carotene, β-cryptoxanthin, lutein, zeaxanthin, and lycopene being the most common dietary carotenoids [100] (Figure 5). Their main biological activities are related to cardiovascular diseases risk reduction and mentaining optimal visual function maintaining [101]. However, besides these systemic effects, their consumption has been considered beneficious for skin health, especially for photoprotection purposes shown by most of the following human studies.

Several studies have demonstrated the capacity of carotenoids’ supplements to prevent UV-induced skin damage in human volunteers. One study demonstrated the effects of the consumption for 6 weeks of a specific probiotic (Skin-Probiotic^TM^) and carotenoids in decreasing early UV-induced skin damage as well as in modulating early skin biomarkers of UV effects [102]. Three different studies using a total of 139 subjects and exposing subjects to UVA, solar radiation and natural sunlight exhibited statistically significant differences (*p* < 0.05) in MED, skin damage biomarkers and skin colour by comparing subjects before and after treatment [102].

Lee et al. investigated the photoprotective effects of a 30 mg carotenoid mixture (29.4 mg β-carotene, 0.36 mg α-carotene, 0.054 mg lutein) per day in a limited number of subjects (11 men and 11 women) for 8 weeks, but no placebo group was used. The concentration of carotenoid supplement was enhanced at 30 mg increments to a final dose of 90 mg per day. The results showed a modest, but significant dose-dependent increase in MED with 60 and 90 mg carotenoid per day (*p* < 0.05). In addition, serum β-carotene concentrations increased after each intake, and serum lipid peroxidation was reduced, although no β-carotene was found in the skin [103].

In a study by Gollnick et al., 20 healthy young female students received a moderate dose of β-carotene, 30 mg per day for 10 weeks. After the supplementation period, supplementation continued with exposure to natural sunlight for 13 days. An increased yellow pigmentation of skin surfaces and a reduction of erythema in subjects that had taken β-carotene was observed. The study concluded that pre-supplementation with moderate dose of β-carotene before and during sun exposure protects against sunburn by significantly decreasing erythema and increasing Langerhans cells (*p* < 0.01). Results also showed that the combination of systemic and topical photoprotection by sunscreens offers a synergistic effect, effect but the number of subjects was limited and no placebo group was used [104].

Postaire et al. also investigated the beneficial effects of a combination of β-carotene and other antioxidants. In this study, a limited number of subjects (10) received a supplement providing 13 mg β-carotene, 2 mg lycopene, 5 mg tocopherol and 30 mg ascorbic acid per day for 8 weeks. Although photoprotection was not directly measured in the study, authors suggest that carotenoids may be photoprotective because of a melanogenesis stimulation [105].

In another study, 20 subjects received 25 mg of carotenoid mix (23.8 mg β-carotene, 0.75 mg α-carotene, 0.18 mg cryptoxanthin, 0.15 mg zeaxanthin and 0.12 mg lutein) per day and another group received this mix and 335 mg α-tocopherol for 12 weeks. After this period, both groups showed a yellowing of the skin and elevated concentrations of β-carotene in serum and skin. The degree of erythema was highest in the group that received the carotenoids mix only (*p* < 0.01), so, the authors concluded that vitamin E might increases carotenoid protection against UV irradiation [106].

The effect of a mixture of the three main dietary carotenoids, beta-carotene, lutein and lycopene (8 mg/day each), compared to beta-carotene (24 mg/day from an algal source) was evaluated in the erythema-protective effect. Thirty-six subjects were randomly assigned to three groups of 12 subjects in a placebo-controlled, parallel study design. The first group of treatment received 24 mg of β-carotene per day for 12 weeks, the second group received the carotenoid mix and the third group received placebo. Although the number of subjects was low, the results showed that the intensity of erythema 24 h after irradiation was significantly decreased in both groups treated with carotenoids after 12 weeks of treatment, in correlation with the increase of serum carotenoids (*p* < 0.001). An increase of serum and skin concentration of carotenoids was determined in groups 1 and 2 throughout the study. Therefore, both carotenoid based treatments ameliorated UV-induced erythema in humans in a similar way [107].

## 6. Vitamin Supplementation

Vitamins are organic compounds which are essential nutrients for humans. They are required in limited amounts from diet and belong to different structural families. Vitamins also play a significant role in skin health [108], exerting different actions including antioxidant activity, sebum and keratinisation regulation, collagen synthesis, ECM homeostasis and photoprotection [108]. Several nutraceutical products containing vitamins as their main ingredient have been tested in humans as detailed below. 

A mixture of vitamins derived from fermented papaya (*Carica papaya* L.) and an antioxidant cocktail was tested in 60 healthy subjects. Subjects received 4.5 g per day of fermented papaya preparation (FPP, final composition per 100 g: 90.7 g carbohydrates, 17 μg vitamin B6, 2 μg folic acid, 2.5 mg calcium, 16.9 mg potassium, 240 μg niacin, 4.6 mg magnesium, 14 μg copper, 75 μg zinc, 16 mg arginine, 6 mg lysine, 5 mg histidine, 11 mg phenylalanine, 9 mg tyrosine, 18 mg leucine, 9 mg isoleucine, 5 mg methionine, 13 mg valine, 11 mg glycine, 8 mg proline, 37 mg glutamic acid, 11 mg serine, 8 mg threonine, 27 mg aspartic acid, and 2 mg tryptophan) and an antioxidant cocktail (10 mg trans-resveratrol, 60 μg selenium, 10 mg vitamin E and 50 mg vitamin C) for 90 days. Results showed a significant improvement in skin elasticity, moisture and antioxidant capacity (*p* < 0.05) with both the papaya preparation and the antioxidant cocktail [109].

In another study, 33 subjects received 100 or 180 mg per day of vitamin C or placebo for 4 weeks. After treatment, results showed that 100 mg of orally administered vitamin C increased radical scavenging activity of the skin in 22% while 180 mg dose increased antioxidant activity by 37%, compared to baseline [110].

In a double-blind trial, 16 healthy subjects received vitamins and trace elements to investigate the photoprotective effects of these compounds. The first group received 200 μg selenium, 16 mg copper sulfate, 14 mg α-tocopherol and 2700 μg retinol; the second group only received trace elements (200 μg selenium and 16 mg copper sulfate); the third group received only vitamins (14 mg α-tocopherol and 2700 μg retinol) and the last group received placebo for 3 weeks. Supplementation in all treatments with active elements showed protection against sunburn cells at a low UV irradiation dose compared with the placebo group, but total number of subjects was very limited for three groups. However, treatment with both trace elements and vitamins showed an additional reduction in the number of sunburn cells [111].

The photoprotective effects of vitamins E and C have been intensely studied. A combination of vitamins E and C showed a protective effect in a double-blind, placebo-controlled study with 20 subjects. Ten subjects received 2 g of vitamin C and 1000 IU of vitamin E per day or placebo for 8 days. The sunburn reaction before and after 8 days of the treatment was assessed by MED and by measuring the cutaneous blood flow of irradiated skin. Combined vitamin C and E reduce the sunburn reaction, UV-induced skin damage and cutaneous blood flow, whereas it increased in the placebo group [112]. However, the low number of subjects provided a low statistical resolution.

Fuchs and Kern also demonstrated a photoprotective effect of a combination of vitamins E and [113]. In this study, 40 healthy subjects received 2 g vitamin E per day, 3 g vitamin C per day, a combination of both vitamins and placebo for 50 days, so only 10 subjects were used per group. MED increased after intake slightly in subjects who received either vitamin alone or placebo. Nevertheless, the combination of both vitamins showed more pronounced photoprotective effect. Authors suggest that vitamin E and C act synergistically in suppression of sunburn reaction [113].

Placzek et al. investigated the effect of long-term oral administration of a combination of vitamin C and vitamin E in subjects on UVB-induced epidermal damage. Eighteen volunteers (12 males and 6 females) received supplementation with 2 g vitamin C and 1000 IU vitamin E per day for 3 months. Blood vitamin concentrations were measured at the beginning and every 30 days during the study. After 3 months of treatment, the intake of vitamin C and vitamin E significantly reduced sunburn reaction to UVB irradiation. In addition, thymine dimers induced by UVB irradiation present in the skin was significantly reduced [114]. Again, the low number of subjects per group provided low statistical resolution. 

## 7. Coenzyme Q10 (CoQ10)

CoQ10 is a natural compound of food and is often used in both functional foods and supplements. It is an endogenous lipophilic compound, essential component of mitochondrial energy metabolism, an effective antioxidant and presents a range of putative benefits for human health [115,116,117,118]. In a double-blind, placebo-controlled study, 33 healthy subjects received 50 mg or 150 mg CoQ10 (Q10Vital^®^), or placebo for 12 weeks. Results did not show significant changes in the MED. However, supplementation with CoQ10 limited deterioration of viscoelasticity (*p* < 0.05) and decreased some visible signs of ageing such as wrinkles and micro-relief lines (*p* < 0.05) and improved skin smoothness [119].

## 8. Polyunsaturated Fatty Acids (PUFAs)

PUFAs have been traditionally considered beneficious for human health [120,121] and many health agencies and institutions have recommended their consumption. Structurally, PUFAs are divided into an omega-3 and an omega-6 series, depending on the position of their double bonds [122]. Their biological activity is especially relevant to cardiovascular and other inflammatory diseases, but also over skin heath [123,124,125,126]. Some studies have been developed in humans using PUFAs-based nutraceutical formulations as described below. However, most studies used a low-to-moderate number of subjects that result in low statistical resolution.

To test the photoprotective capacity of fish oil, ten subjects supplemented their diets with ten capsules per day of fish oil containing each 280 mg eicosapentaenoic acid (EPA) and 120 mg docosahexaenoic acid (DHA), and 10 subjects received ten placebo capsules per day. After 2 weeks, no significant effect of fish oil supplementation was observed in the measured parameters. However, after 4 weeks a small statistically significant increase of MED was seen in the fish oil treated group. These authors conclude that a low dose of fish oil (EPA and DHA) in a short period may be photoprotective [127]. In a study by Rhodes et al., 15 female Caucasian subjects supplemented their diets with 10 g of fish oil per day (18% EPA and 12% DHA) for 6 months. After 6 weeks of treatment, an increase in MED was observed but at 10 weeks MED decreased again. Although fish-oil intake reduced UV-induced erythema, the lipid peroxidation of skin increased due to the unstable nature of *n*-3 fatty acids [128]. In another study, the protective effect of EPA supplementation was investigated. For that purpose, 28 subjects received 4 g EPA (98%) or oleic acid (98%) per day for 3 months. UVB irradiation-induced erythema and p53 induction decreased in the EPA-treated group while no significant changes were found in the oleic acid group [129].

SemoSqualene^®^, is an oral supplement rich in squalene (Figure 5), a polyunsaturated aliphatic hydrocarbon, and has been used in 40 healthy female subjects (>50 years of age). A high dose of squalene (13.5–27 g per day) for 90 days reduced facial wrinkles, decreased skin reactivity to UV as shown by increased MED, increased type I procollagen gene expression and reduced UV-induced DNA damage and apoptosis. These effects may be attributed to its antioxidant capacity [130].

Several human studies have been performed to test the capacity of fish oil to alleviate eczema or psoriatic lesions. Ziboh et al. determined the benefits of fish oil supplementation in 13 psoriasis patients receiving a supplement ranging from 60 to 75 mg (18% EPA and 12% DHA per gram) for 8 weeks. At the end of treatment, results showed that 8 patients demonstrated mild-to-moderate improvement in their psoriatic lesions which correlated with a high EPA-DHA ratio into the sera, epidermal and neutrophil lipids. Results suggested that increase in the ratio of leukotriene B_5_ (LTB_5_) was responsible for the reduction in inflammation [126]. In a similar study, ten patients with severe chronic psoriasis received 12 g EPA per day for 6 weeks. After treatment, a reduction in erythema was observed in 80% of patients [131]. A much lower dose of fish oil extract which provided 1.8 g EPA (MaxEPA^®^) was administered daily to 28 psoriasis patients for 12 weeks. After 8 and 12 weeks, itching, erythema, and scaling decreased in the active treatment group, with a trend towards an overall decrease in body surface area affected, while no changes occurred in the placebo group [132]. 

Only two studies performed on patients with psoriasis or atopic eczema used a large number of subjects. First, 80 patients with chronic and stable psoriasis were supplemented with 1122 mg EPA and 756 mg DHA for 8 weeks. Results showed an improvement in the overall skin condition and individual disease such as pruritis, scaling, induration and erythema [133]. Second, 99 subjects (60 adults and 39 children) with atopic eczema received primrose seed oil (EPO) for 12 weeks. Adults were supplemented with 1440 mg linolenic acid and 190 mg γ-linolenic acid per day. Children received 720 mg linolenic acid and 90 mg γ-linolenic per day. Results showed a moderate improvement in clinical signs of atopic eczema after treatment [134]. 

## 9. Discussion

The nutraceuticals market focused on skin health is increasing driven by consumer demand but strong scientific evidence for the products is still scarce. On the one hand, the increasing demand of naturally based products points to a promising future and plenty of opportunities for companies. On the other hand, scientific evidence about these products must increase considerably to improve products credibility.

First, although some studies include plasmatic measurements of the main compounds and metabolites derived from the administered formulations, most of the studies did not provide this information. The correlation between plasmatic concentrations of the metabolites and the observed biological effects would allow for the establishment of a cause-and-effect relationship and would improve claim substantiation. Bioavailability studies are deficient for different reasons. In some cases, either the bioavailability of a single pure compound is studied and not that of the same compound in the presence of the total formulation. In other cases, only in vitro absorption studies are performed, which are difficult to extrapolate in vivo. Besides bioavailability studies, mechanistic studies in skin cell models must also be performed to provide with a rationale of the observed biological effects.

Another difficulty when trying to compare different studies is related to the big differences in doses, additives and galenic formulations for the same active ingredient that prevents appropriate comparisons. Formulations are optimised by authors to improve solubility, absorption or bioavailability and to be technologically feasible. Therefore, even when similar doses of the same ingredient are used, a large variability is obtained between studies due to the formulation, besides the different baseline skin characteristics of the subjects.

However, the most important issue of the human trials using nutraceuticals for skin purposes is the low statistical resolution for some of these studies and, therefore, the low significance of the results. In most cases, as shown in this review, differences between treated and placebo groups are statistically significant, but their clinical significance is quite low due to the low number of subjects. In other cases, the biological significance is poor due to measurements based on questionnaires or personal perceptions. Some studies even lack a placebo group, which makes it difficult to counteract other effects such as diet, season or lifestyle. It might be the time for standardisation and including a minimum number of subjects and skin measurements in order to provide adequate statistical resolution when a human trial protocol is submitted to an Ethics committee.

This review tries to compile the most relevant human trials based on nutraceutical products related to skin and cosmetic issues. The human trials reviewed here cover many different ingredients: proteins, oligosaccharides, lipids, vitamins, polyphenols, and also pure ingredients or complex formulations. In any case, the strengths and weaknesses of the reviewed studies must be highlighted in order to be improved in the future.

Some human trials with collagen peptides did not use a placebo group or used a complex mixture into the dietary supplement [74], hence, establishing a cause-effect relationship for the ingredient was difficult. Regarding bioactive polysaccharides, despite statistically significant differences observed in some of the studies performed with Imedeen^®^ ingredient, the small number of subjects utilised and the absence of a placebo group may cause doubts about the results obtained [75,76]. The authors also reported that five patients in the Vivida^®^ group developed transient, mild pimples during the first weeks of treatment. However, trials made with galacto-oligosaccharides seemed to show consistent results on the improvement of hydration and skin barrier function [80].

French maritime Pine bark extract, Pycnogenol^®^, has been studied in at least five different human trials. However, the low to moderate number of subjects provides low statistical resolution, the lack of placebo group in some cases and the inclusion of Pycnogenol^®^ into complex mixtures prevent establishing a cause-effect relationship, suggesting that some of the results on Pycnogenol^®^ ingredient for skin health [87,88,89] must be taken with caution and further research is required. Other studies based on procyanidin-based polyphenolic extracts provided conclusive results on the inhibition of UV-induced age spots due to the high number of subjects utilised [92]. The efficacy of rosemary and citrus polyphenols to reduce skin redness and MED and to improve skin elasticity and decrease wrinkle depth in correlation to a decrease in skin lipid oxidation was also consistently proven [96], and a mechanistic rationale was also provided in skin cell model [95]. A red orange extract enriched in anthocyanins [97] and *Polypodium leucotomos* extract [98,99] also exhibited evidence of improvement in several skin signs related to photo-aging (erythema, hydration, elasticity and pigmentation), but with a much lower number of subjects, which compromises the consistency of the results.

Available studies on nutraceuticals containing carotenoids have shown a modest increase of MED with doses of 30–90 mg/day, but again, too small a number of subjects was used in most studies to be conclusive [103,104,105,106,107]. Human studies on vitamins, especially vitamin E and vitamin C, exhibited photoprotective properties through the increase of skin elasticity, moisture and antioxidant capacity in correlation with a MED increase [109,110,111,112,113,114]. Finally, studies that used PUFAs-based nutraceuticals showed inconsistent results [127,128,129]. Nevertheless, a reduction of erythema and inflammation was noticed in patients with psoriatic lesions or eczema taking omega-3 based nutraceuticals [126,131,132,133].

## 10. Conclusions

A number of natural ingredients have been shown to be potentially effective to alleviate the signs of skin ageing and some skin diseases. The rationale for the putative beneficial effects of an ingredient must be primarily demonstrated in skin cell models to elucidate the possible mechanism of action. Although nutraceuticals are under food regulation, the EU cosmetic legislation (2013/674/EU: Commission Implementing Decision of 25 November 2013) banned in the marketing of cosmetic ingredients or products which have been tested on animals. Therefore, the only options to verify the efficacy of a cosmetic ingredient are either in vitro skin cell models or human trials. To date, available human trials are far from conclusive in many cases for different reasons. In some cases, the ingredient tested is poorly characterised or it is part of a complex mixture that prevents establishing a cause-effect relationship between the ingredient and the biological effects. In other cases, clinical trials show poor statistical power due to insufficient number of subjects or the lack of a placebo group. Moreover, human trials only determine macroscopic skin characteristics (redness, spots, elasticity, wrinkles, etc.) and do not measure skin molecular markers (oxidative stress, enzymatic activity, gene expression or metabolites) that provide a mechanistic base. Therefore, in vitro and human trials on skin nutraceuticals must be designed to overcome all these limitations. Skin nutraceuticals are expected to shortly become a very profitable market. Nevertheless, more and better human trials must be performed in order improve the scientific basis of skin nutraceuticals and their credibility.
